# The immune hypothesis of synesthesia

**DOI:** 10.3389/fnhum.2013.00563

**Published:** 2013-09-11

**Authors:** Duncan A. Carmichael, Julia Simner

**Affiliations:** ^1^Department of Psychology, University of EdinburghEdinburgh, UK; ^2^Institute for Adaptive and Neural Computation, University of EdinburghEdinburgh, UK; ^3^Division of Psychiatry, Royal Edinburgh Hospital, University of EdinburghEdinburgh, UK

**Keywords:** synesthesia, genetics, immunity, neurological basis, genes, cortical connectivity, immune system

Synesthesia is a hereditary, neurological condition in which a wide range of common stimuli (e.g., letters, sounds, flavors) trigger unexpected secondary sensations, for example, synesthetes listening to music might see colors in addition to hearing sounds (Ward et al., 2006; see Simner and Hubbard, 2013, for review). Current explanations of synesthesia posit structural and/or functional differences in the synesthete brains, and frame their models in terms of excess cortical connectivity or altered cortical feedback. Here, we propose an immune hypothesis of synesthesia, which supplements existing models by suggesting how such altered connectivity may arise and how associations between synesthesia and other conditions might be explained.

Two categories of model seek to explain the generation of synesthetic experiences, and more recent models are a hybrid of both (Brang et al., 2010). The *cross-activation model* (Ramachandran and Hubbard, 2001) suggests that excess connectivity between functional areas of the cortex allows activation in one cortical area (e.g., auditory cortex) to directly trigger activation in another (e.g., visual cortex). Evidence in support of this model comes for example from diffusion tensor imaging (DTI) and shows that excessive connectivity is indeed a feature of the synesthetic brain (Rouw and Scholte, 2007). *Re-entrant* and *disinhibited feedback models* propose that synesthetic sensations are caused by disinhibited feedback from higher cortical areas (e.g., in parietal lobe) failing to suppress non-relevant activation from lower cortical areas (Grossenbacher and Lovelace, 2001). This type of disinhibited feedback may result from excessive activity of excitatory neurons within the delicate balance between both excitatory and inhibitory neurons in the brain (Hubbard et al., 2011). Despite appearing superficially different, connectivity and feedback models need not be mutually exclusive. It is unlikely that altered feedback happens entirely in the absence of changes in cortical connectivity, given the Hebbian principle that simultaneous activity strengthens interconnectivity between neurons. Therefore, these two approaches might be considered somewhat unified in that connectivity models propose aberrant connectivity as the primary causal mechanism underlying synesthesia whereas feedback models might allow altered connectivity as an *indirect* consequence of disinhibited feedback.

While these models are now more than a decade old, explanations of *how* these cortical characteristics might arise have proven elusive thus far (but see Brang and Ramachandran, 2008; Mitchell, 2013) and we explore this here. Synesthesia is thought to be primarily neurodevelopmental in nature (Spector and Maurer, 2009). Consequentially, known processes of brain development are likely to be implicated in its emergence. We propose that insight might be gained from examining the functionality of genes that regulate the types of altered synesthetic cortical connectivity assumed in these models above (i.e., genes for axon guidance, synapse density). This is the approach we follow here.

## Dual gene functionality: connectivity and immunity

Above, we saw that current models link synesthesia to altered structural connectivity, misregulated feedback mechanisms, or a combination thereof. Explaining how synesthesia develops might therefore come from considering the developmental processes responsible for cortical connectivity. The immune system is known to play a significant role in these processes (for review, see Boulanger, 2009) and we ask here whether a propensity to develop synesthesia may be linked to the expression of immune proteins in the CNS, since this expression can be conferred by genes with functions of both immunity and cortical development. Indeed, many genes have been shown to have precisely this dual functionality (Boulanger, 2009). As well as their immunity function, such genes act in cortical development, altering structural and/or functional connectivity by influencing the development of axonal guidance, synaptic connectivity, and synaptic pruning. One outcome of these changes may therefore be the anomalous pattern of connectivity proposed by the cross-activation theory in the development of synesthesia. Alternatively, the immune system could have a direct influence on excitatory neuronal activity, leading to the outcomes proposed by disinhibited feedback models. This is because the immune system plays an important role in the initial development and subsequent plasticity of glutamatergic synapses, the primary excitatory transmission pathway in the mammalian cortex (Fourgeaud and Boulanger, 2010).

## Can the immune system influence the development of the brain?

Is it plausible to make a link between the immune system and regulation of the central nervous system (CNS)? Isolated from the rest of the body by the blood-brain-barrier, the CNS was once thought to barely interact with the immune system, leading to the long held view that the CNS was “immune privileged” (McAllister and van de Water, 2009). However, research now shows a complex communication between the CNS and immune system, with wide-reaching consequences for brain regulation and development, both in health and disease (Elmer and McAllister, 2012). Immune proteins are known to play a role at many stages in the developmental pathway. They are integral components of phases critical to brain development and plasticity, such as neuronal guidance, synapse development, and synaptic remodeling (Boulanger, 2009). We therefore hypothesize that CNS and immune system interaction may be the biological mechanism which confers the predisposition to develop synesthesia.

Which aspects of the immune system are known to exhibit the type of dual functionality under discussion in this article (i.e., functionality in both immunity and cortical development)? Several areas of this extraordinarily complicated system are worth highlighting. The *complement system* is one possible candidate—a complex cascade of protein interactions involved in immunity which has also been shown to play an important role in tagging of synapses to be eliminated by pruning during development (Stephan et al., 2012). Another candidate relates to cytokines, which are immune proteins that have also been shown to play significant roles in neurogenesis and synaptic plasticity (Bauer et al., 2007). A third candidate relates to major histocompatibility complex (MHC) proteins, which are an integral part of the adaptive immune system found on the surface of the majority of nucleated cells and widely expressed in neurons of the CNS (Boulanger, 2004). In addition to fulfilling a crucial function in immune response, MHC class I molecules and related components are thought to be involved in a range of developmental processes, such as activity-dependent plasticity and synaptic refinement (Boulanger, 2009). The MHC locus contains several hundred genes, and has also been widely implicated in a range of autoimmune conditions, such as multiple sclerosis (MS), irritable bowel syndrome (IBS), and rheumatoid arthritis (Fernando et al., 2008). We point out, however that the immune system consists of many hundreds of individual factors and processes. Given this, our suggestions above should be considered speculative and by no means exhaustive. Nevertheless, we consider them to each be plausible candidates for future investigation.

We end this section by asking whether existing studies into the genetics of synesthesia would support our immunity hypothesis. In other words, have they identified areas of the genome containing immune system genes? Research into synesthesia genetics is in its infancy and as yet, there are insufficient data to draw firm conclusions. No synesthesia genes have yet been identified and no firm mode of inheritance has yet been elucidated. However, evidence from the two existing studies on the genetics of synesthesia (Asher et al., 2009; Tomson et al., 2011) have identified several chromosomal regions of interest, and these regions do contain immune function genes. Asher et al. (2009) found significant linkage to chromosome 2q24 and possible linkage to areas on other chromosomes (5q33, 6p12, and 12p12), while Tomson et al. (2011) identified a candidate region on chromosome 16q12.2-23.1. The authors of both studies draw the conclusion that synesthesia is likely to be a condition influenced by a variety of genes in multiple loci. Nonetheless, the chromosomal regions of interest highlighted in these two investigations do contain immune function genes (e.g., interleukin-17, a cytokine protein found on chromosome 6p12), although we wish to be clear that many other viable candidates also lie outwith these regions.

## The immune hypothesis as a framework for the study of co-morbidities

An immune hypothesis of synesthesia might additionally explain recent co-morbidity data which suggests that having synesthesia may be associated with increased risk of other clinical conditions. Carruthers et al. (2012) report an association between synesthesia and IBS, having found an elevated prevalence of synesthesia in a population of people with IBS. Other researchers have raised the possibility that synesthesia may also be found at elevated rates within populations with autism (Baron-Cohen et al., 2007) or migraine (Alstadhaug and Benjaminsen, 2010). The immune system plays a prominent role in all of these conditions (Collins, 2002; Bruno et al., 2007; Enstrom et al., 2009), suggesting that altered immune system function may be a common causal link. If so, the immune model proposes a plausible framework by which to investigate co-morbidity between synesthesia and other conditions. If this hypothesis is correct, we might ask whether the prevalence of synaesthesia is also higher in populations with other autoimmune conditions. Indeed, recent data from our lab has led us to explore whether developmental synaesthesia might occur more prevalently in people with the radiological profile of multiple sclerosis (MS), for example, a demyelinating disease of the human CNS (Simner et al., submitted). A maladaptive immune system is an undisputed factor in the pathogenesis of MS (Trapp and Nave, 2008), and the majority of genes implicated in MS have an immune function (Gourraud et al., 2012). The immune hypothesis of synaesthesia might therefore lead us to investigate whether synaesthesia and autoimmune conditions such as MS could share overlapping genetic origins in contributing to cortical development and immune function.

## Conclusion and future directions

We have proposed that CNS/immune system interactions during early life may play a role in the development of synesthesia. We have asked whether genes with dual functionality in brain development and immunity may be at the origin of existing models of synesthesia, and this mechanism would provide a framework to investigate associations between synesthesia and other immune-related conditions. We make our proposal here as a model for developmental synesthesia, although not all cases of synesthesia are developmental in nature. Synesthesia may also be acquired, for example as a result of brain injury (Schweizer et al., 2013) or induced by consumption of psychoactive drugs such as Lysergic acid diethylamide (LSD; e.g., Cytowic, 1989). Our hypothesis does not speak directly to such cases, and it is not yet known whether these different forms of synesthesia have the same neural origins or mechanisms. It is interesting to note however that immune system activity is elevated after brain injury, and processes such as apoptosis do become activated (Griffiths et al., 2010). It is therefore at least plausible to ask whether the immune system might also play a role in the appearance of non-developmental synesthesias. Identification of genes that contribute to the development of synesthesia will make a significant contribution to the validity of this hypothesis, and whether synesthesia has one cause or many.
